# Efficacy of mesenchymal stem cells in animal models of lupus nephritis: a meta-analysis

**DOI:** 10.1186/s13287-019-1538-9

**Published:** 2020-02-04

**Authors:** Tianbiao Zhou, Chunling Liao, Hong-Yan Li, Wenshan Lin, Shujun Lin, Hongzhen Zhong

**Affiliations:** 1 Department of Nephrology, the Second Affiliated Hospital, Shantou University Medical College, 515041, No. 69 Dongsha Road, Shantou, China; 2 Department of Nephrology, Huadu District People’s Hospital of Guangzhou, Southern Medical University, Guangzhou, 510800 China

**Keywords:** Systemic lupus erythematosus (SLE), Lupus nephritis, Mesenchymal stem cells, Efficacy, Meta-analysis

## Abstract

**Background:**

Lupus nephritis is usually manifested by proteinuria, active urinary sediment, hypertension, and renal failure and is a serious complication with more than 50% occurrence in systemic lupus erythematosus patients. Mesenchymal stem cells (MSC) present remarkable immunomodulatory ability, and these cells are potential therapeutic agents for autoimmune disorders. In clinical trials, the effectiveness of MSC in the treatment of lupus nephritis is still controversial. A meta-analysis was performed to assess whether MSC can achieve good efficacy in the treatment of lupus nephritis in mice.

**Methods:**

A comprehensive literature search was performed in Cochrane Library, ISI Web of Science, PubMed, and EMBASE from inception to Oct 1, 2019. Two authors independently extracted the data, which were pooled and calculated using RevMan 5.3.

**Results:**

A total of 28 studies met the inclusion criteria. MSC treatment resulted in lower levels of ds-DNA (OR = − 29.58, 95% CI − 29.58, − 17.99; *P* < 0.00001), ANA (OR = − 70.93, 95% CI − 104.55, − 37.32; *P* < 0.0001), Scr (OR = − 8.20, 95% CI − 12.71, − 3.69; *P* = 0.0004), BUN (OR = − 14.57, 95% CI − 20.50, − 8.64; *P* < 0.00001), proteinuria (OR = − 4.26, 95% CI − 5.15 to − 3.37; *P* < 0.00001), and renal sclerosis score (OR = − 1.92, 95% CI − 2.66 to − 1.18; *P* < 0.00001), and MSC treatment could get higher levels of albumin. To detect the potential, the cytokines were also assessed, and the MSC treatment group had lower levels of IL-2, IL-12, IL-17, and IFN-γ when compared with the control group. However, the difference was not notable for IL-4, IL-6, IL-10, TGF-β, MCP-1, TNF-α, Th1, Th17, Foxp3, or Tregs.

**Conclusion:**

Our study confirmed that MSC treatment in an animal model for lupus nephritis in the studies included in the meta-analysis resulted in lower levels of ds-DNA, ANA, Scr, BUN, proteinuria, and renal sclerosis score, and MSC treatment could get higher levels of albumin.

**Electronic supplementary material:**

The online version of this article (10.1186/s13287-019-1538-9) contains supplementary material, which is available to authorized users.

## Introduction

Systemic lupus erythematosus (SLE) is a typical autoimmune disease characterised by the production of autoantibodies against nuclear antigens, which is associated with multiple organ manifestations including lupus nephritis [1]. Lupus nephritis is usually manifested by proteinuria, active urinary sediment, hypertension, and renal failure and is a serious complication with more than 50% occurrence in SLE patients [2]. The causes of many individual SLE pathologies are poorly understood. Traditional therapy includes glucocorticoids, cyclophosphamide, cyclosporine A, tacrolimus, mycophenolate mofetil, azathioprine, etc. However, toxic side effects are notable in some of the drugs mentioned above. Mesenchymal stem cells (MSC) are used to treat some autoimmune diseases and are considered a safer agent when patients are resistant to these conventional therapies mentioned above [3].

MSC are multipotent cells, and they have the capacity to self-renew and differentiate into the tissues of mesodermal origin [4, 5]. They present immunomodulatory ability and are used as therapeutic agents for autoimmune disorders [4, 6, 7]. In clinical trials, the efficacy of MSC in the treatment of lupus nephritis is still controversial. The reason might be that there were many confounding factors among the patients in the studies, such as varying disease severity, different complications and the drugs used in combination. Furthermore, because 10 MSC products are currently approved globally and only 2 are used for immune modulatory effects in host vs graft reactions in humans [8, 9], there are rare confounding factors in studies on animals in vivo. In this study, we included studies on mice to assess the efficacy of MSC in the therapy of lupus nephritis in mice, in order to draw a more robust result for the effectiveness of MSC treatment for lupus nephritis.

## Materials and methods

### Search strategy

A comprehensive literature search, restricted to English-language literature, was performed in the Cochrane Library, Embase, ISI Web of Science, and PubMed databases up to Oct. 1, 2019, using the following search terms: (stem cells OR mesenchymal stem cells OR mesenchymal stromal cells OR multipotent stromal cells OR mesenchymal progenitor cells) AND (systemic lupus erythematosus OR SLE OR lupus nephritis OR LN). The references cited in the recruited articles were also checked to identify additional reports. The Preferred Reporting Items for Systematic Reviews and Meta-Analyses (PRISMA) checklist is presented in Additional file 1.

### Inclusion and exclusion criteria

Inclusion criteria are as follows: (1) type of study: animal experiment that used mice, (2) object of the study: lupus nephritis; (3) interventions: MSC for treatment; and (4) outcome: efficacy.

Exclusion criteria are as follows: (1) reviews, case reports, letters, clinical studies, systematic reviews, and meta-analysis; (2) studies lacked the targeted indicators and were conducted in humans; and (3) the therapeutic regimen included other agents with unknown effects.

### Outcome measures

The following data regarding the efficacy of MSC treatment were identified from the recruited investigations: ds-DNA, ANA, Scr, BUN, albumin, proteinuria, IgM, Foxp3, IL-2, IL-4, IL-6, IL-10, IL-12, IL-17, TGF-β, MCP-1, IFN-γ, TNF-α, Th1, Th17, Tregs, and renal sclerosis score. When there were multiple groups for the MSC-treated group, we only included the data from the early treatment group. When disagreements happened, a discussion with a third reviewer was conducted to resolve it.

### Quality assessment

The methodological quality was independently assessed by two investigators (Tianbiao Zhou and Chunling Liao) using the Cochrane Handbook for Interventions. The principal components used for the assessment of each investigation included attrition bias, detection bias, selection bias, reporting bias, and other bias. Each item was classified as low risk, high risk, or unclear, and the general risk of bias was determined by taking all items together for presentation in a risk bias graph.

### Statistical analysis

We conducted a meta-analysis of all animal studies in mice using the data from the MSC therapy group and control group. Review Manager Version 5.3 and STATA 12.0 were used to calculate the results. Heterogeneity due to study variation was quantified using *I*^2^ statistics. A fixed effect model was applied if the *p* value was ≥ 0.1, based on the test of heterogeneity. Otherwise, a random effects model was applied to pool the results. Weighted mean differences (WMDs) were used to express the continuous data, and 95% confidence intervals (95% CI) were tested for the recruited investigations with the Mantel-Haenszel (M-H) method. Sensitivity analysis was performed for studies with a total number of test animals less than 16. Publication bias was also tested by STATA software 12.0, using both Egger’s linear regression method and Begg’s rank correlation test. A *p* value < 0.05 was considered statistically significant.

## Results

### Search results

**Fig. 1:**
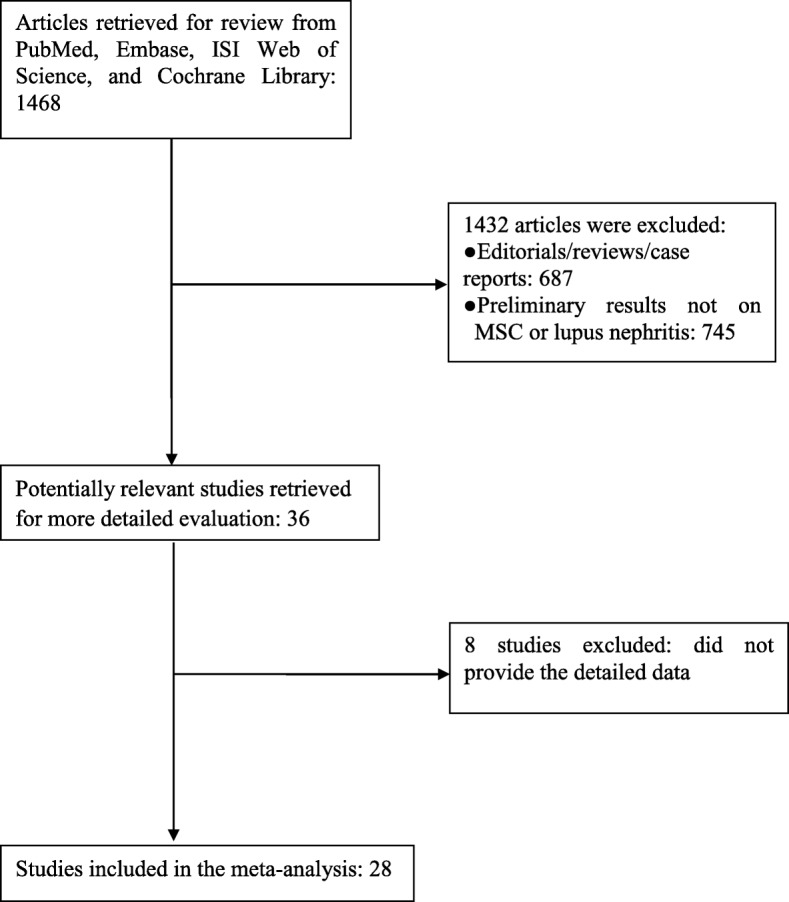
Flow diagram of the selection process

In this meta-analysis, the databases were searched and we only included studies in mice to assess the efficacy of MSC treatment in lupus nephritis. The flowchart for this process is shown in Fig. 1. The characteristics of the recruited investigations are presented in Table 1.

**Table 1:** Characteristics of the studies included in this meta-analysis

Author, year	*n*	Type of animal	MSC type	Number of MSC	Route of delivery	Endpoints
Sun 2009 [10]	12	MRL/lpr mice	BM-MSC	0.1 × 10^6^ cells per 10 g body weight	Intravenous	ds-DNA, albumin, proteinuria, IgM, Foxp3, IL-6, IL-17
Gu 2010 [11]	8	MRL/lpr mice	UC-MSC	1 × 10^6^; multi-treatment (at the 18th, 19th, and 20th weeks of age)	Intravenous	Proteinuria, BUN, Scr, ds-DNA, MCP-1, Foxp3
Chang 2011 [12]	8	NZB/W F1 mice	UC-MSC	1 × 10^6^	Intravenous	Proteinuria, Scr, ds-DNA, IFN-γ, TNF-α, IL-2, IL-6, IL-12, IL-4, IL-10, renal sclerosis score
Choi 2012 [13]	26	NZB/W F1 mice	AD-MSC	1.4 × 10^7^	Intravenous	Proteinuria, BUN, Scr, ds-DNA
Ji 2012 [14]	10	MRL/lpr mice	BM-MSC	0.2 × 10^6^ cells per 10 g body	Intravenous	ds-DNA, ANA, proteinuria
Ma 2013 [15]	9	MRL/lpr mice	BM-MSC	1 × 10^6^	Intravenous	Proteinuria, ds-DNA
Li 2013 [16]	7	129X1/svj mice	BM-MSC	1 × 10^6^	Intravenous	Proteinuria, BUN, renal sclerosis score, MCP-1
Liu 2014 [17]	4	MRL/lpr mice	BM-MSC	1 × 10^6^	Intravenous	Proteinuria, Scr
Che 2014 [18]	10	MRL/lpr mice	BM-MSC	0.1 × 10^6^ cells per 10 g body	Intravenous	ds-DNA, ANA, IL-10, TGF-β
Park 2015 [19]	6	Roquinsan/san mice	AD-MSC	1 × 10^6^ for 5 weeks	Intravenous	IgM, ds-DNA, Th1, Th17, Treg
Choi 2015 [20]	15	MRL/lpr mice	AD-MSC	1 × 10^6^	Intravenous	Proteinuria, MCP-1, TNF-α, IL-2, IL-4, IL-6, IL-10, IL-12, IL-15, IL-17
Jang 2016 [21]	8	NZB/W mice	BM-MSC	1 × 10^6^ for 5 weeks	Intravenous	ds-DNA, proteinuria
Choi 2016 [22]	15	NZB/W mice	AD-MSC	5 × 10^5^ for 27 times	Intravenous	BUN, ds-DNA
Yuan 2016 [23]	16	MRL/Lpr mice	human early embryonic MSC (hMSC)	1 × 10^6^	Intravenous	ds-DNA, albumin, proteinuria, Scr, BUN, IL-17, IL-10, TGF-β
He 2016 [24]	6	B6.MRL/lpr mice	AD-MSC	1 × 10^6^	Intravenous	ds-DNA, proteinuria, IL-17, IL-6, INF-γ, TGF-β, TNF-α
Choi 2016 [25]	20	C3.MRL-Faslpr/J mice	AD-MSC	1 × 10^6^	Intravenous	ds-DNA, Scr, BUN, Treg, Th1
Zhang 2017 [26]	5	B6.MRL-Faslpr mice	UC-MSC	1 × 10^6^	Intravenous	ds-DNA, Th17, renal sclerosis score
Lee 2017 [27]	5	MRL-Faslpr mice	BM-MSC	4 × 10^6^	Intravenous	ds-DNA, proteinuria
Yang 2018 [28]	6	MRL/lpr mice	BM-MSC	2 × 10^6^	Intravenous	Renal sclerosis score, proteinuria, ds-DNA
Tani 2017 [29]	5	NZB/W F1 mice	BM-MSC	1 × 10^6^	Intravenous	Proteinuria, ds-DNA, renal sclerosis score
Mai 2018 [30]	4	MRL/lpr mice	UC-MSC	1 × 10^6^	Intravenous	ds-DNA, proteinuria, IFN-γ, TGF-β, MCP-1, IgM, IL-2, IL-10
Ma 2018 [31]	8	MRL/lpr mice	UC-MSC	1 × 10^6^	Intravenous	Scr, proteinuria, IgM, C3,
Zhang 2019 [32]	3	B6.MRL-Faslpr mice	UC-MSC	1 × 10^6^	Intravenous	Renal sclerosis score, proteinuria, ds-DNA
Lee JH 2018 [33]	6	MRL-Faslpr mice	BM-MSC	4 × 10^4^	Intravenous	ds-DNA, proteinuria
Huang 2018 [34]	12	MRL-Faslpr mice	UC-MSC	1 × 10^6^	Intravenous	Renal sclerosis score, ds-DNA, proteinuria
Liu 2019 [35]	10	B6.MRL-Faslpr mice	placenta-derived mesenchymal stem cells (pMSC)	1 × 10^6^	Intravenous	ds-DNA, proteinuria, TNF-α
Tang 2019 [36]	10	B6.MRL-Faslpr mice	UC-MSC	2 × 10^5^ per 10 g body	Intravenous	ds-DNA, proteinuria, ANA, IgG, IgM, renal sclerosis score, IL-6, IL-17, IL-10, MCP-1

### Quality assessment

**Fig. 2:**
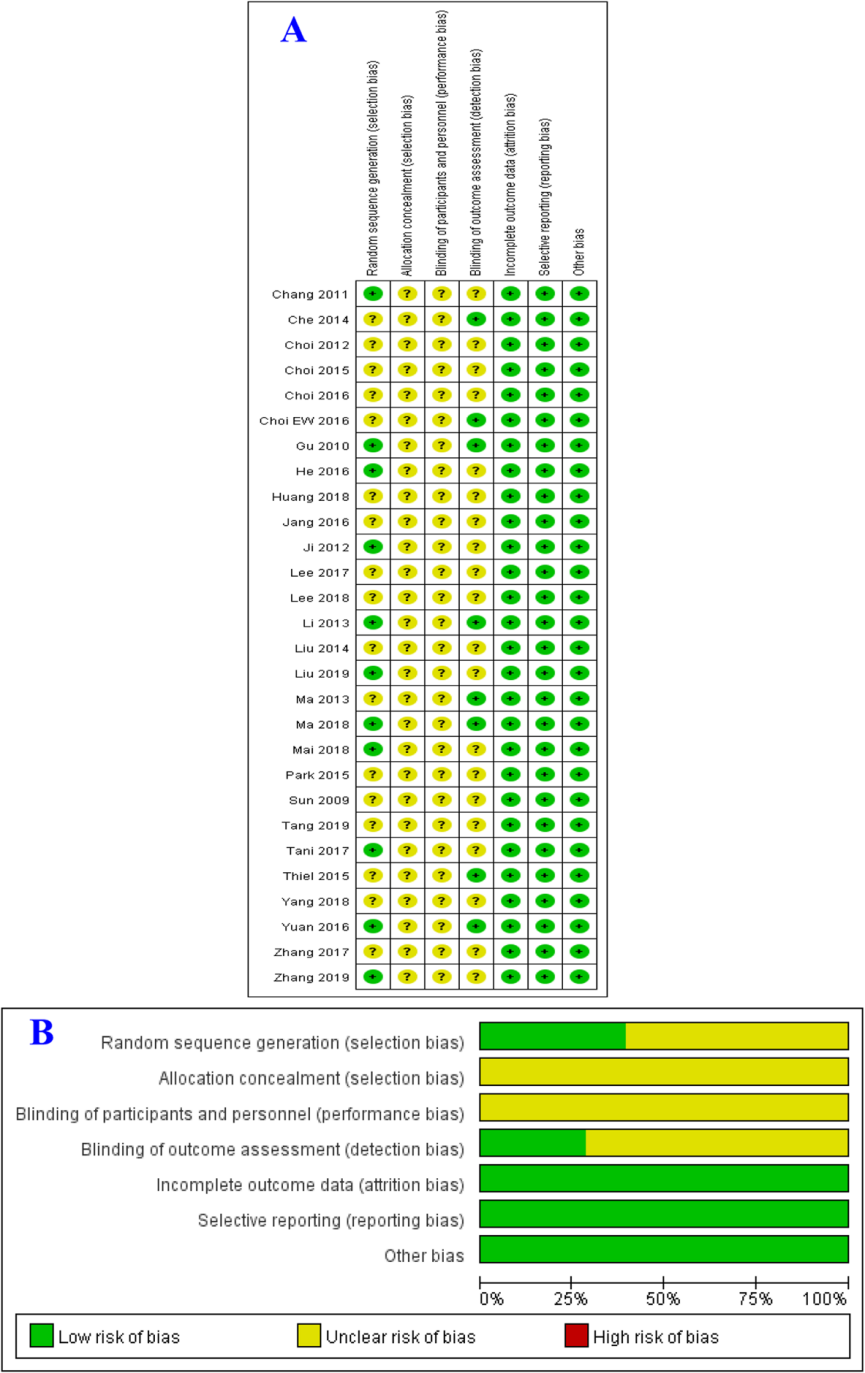
**a** Aggregate risk of bias graph for each experimental animal studies. **b** Risk of bias summary

The methodological quality of the included studies was regarded as acceptable, as most of the domains of the included studies were ranked as low or unclear risk of bias. Low risk of bias was mostly detected in selection bias, detection bias, reporting bias, and attrition bias. Unclear risk of bias mostly occurred in selection bias, performance bias, and detection bias. A summary of the risk of biases of the included studies is presented in Fig. 2.

### Assessment of ds-DNA levels

**Fig. 3:**
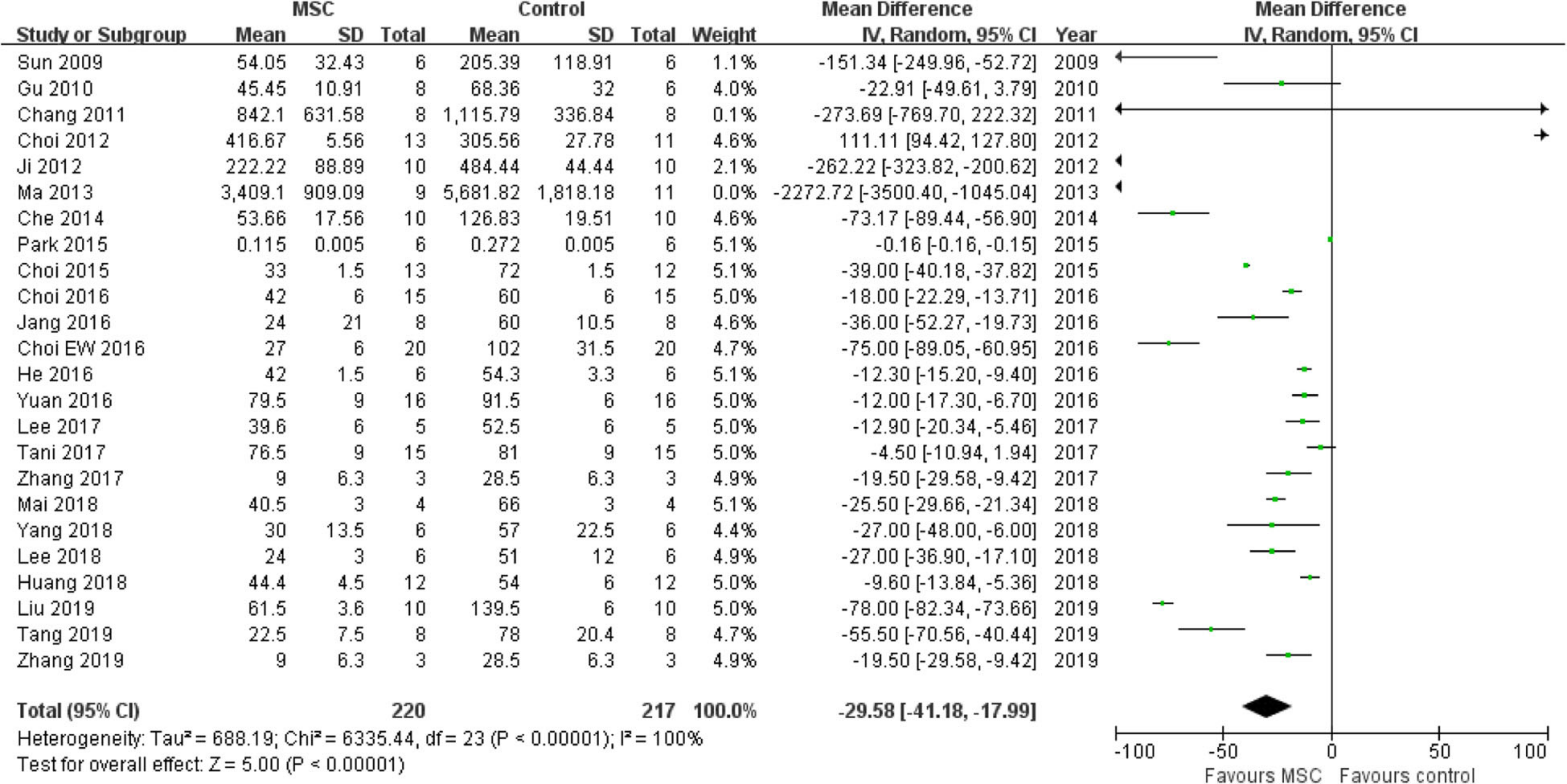
Assessment of ds-DNA levels

Twenty-four studies [10–15, 18–30, 32–35, 36] were recruited. We extracted the data for further analysis, and the results showed that the MSC treatment group obtained a lower level of ds-DNA when compared with the non-MSC treatment group in lupus nephritis mice (OR = − 29.58, 95% CI − 29.58, − 17.99; *P* < 0.00001; Table 2 and Fig. 3).

**Table 2:** Meta-analysis of the efficacy of MSC in the therapy of lupus nephritis

Indicators	Studies	Q test	Model	OR/WMD	*p*
	Number	*p* value	selected	(95%CI)	
ds-DNA	24	< 0.00001	Random	− 29.58 (−41.18, − 17.99)	< 0.00001
ANA	4	< 0.00001	Random	− 70.93 (−104.55, − 37.32)	< 0.0001
Scr	8	< 0.00001	Random	− 8.20 (−12.71, − 3.69)	0.0004
BUN	7	< 0.00001	Random	− 14.57 (−20.50, − 8.64)	< 0.00001
Albumin	2	0.10	Random	7.22 (3.74, 10.69)	< 0.0001
Proteinuria	21	< 0.00001	Random	− 4.26 (−5.15, − 3.37)	< 0.00001
IgM	3	< 0.00001	Random	− 4437.90 (− 12,581.07, 3705.28)	0.29
IL-2	4	< 0.00001	Random	− 50.86 (− 78.76, − 22.96)	0.0004
IL-4	2	< 0.0001	Random	− 92.42 (− 332.33, 147.49)	0.45
IL-6	7	< 0.00001	Random	− 33.55 (− 83.31, 16.21)	0.19
IL-10	6	< 0.00001	Random	− 29.67 (− 68.25, 8.91)	0.13
IL-12	3	< 0.00001	Random	− 328.24 (− 652.20, − 4.29)	0.05
IL-17	5	< 0.00001	Random	− 36.40 (− 65.88, − 6.93)	0.02
TGF-β	3	< 0.00001	Random	− 0.09 (− 2.90, 2.72)	0.95
MCP-1	2	< 0.00001	Random	− 5917.71 (− 17,303.66, 5468.23)	0.31
IFN-γ	4	< 0.00001	Random	− 240.24 (−364.73, − 115.75)	0.0002
TNF-α	6	< 0.00001	Random	− 74.71 (− 167.69, 18.28)	0.12
Th1	3	< 0.00001	Random	− 6.37 (− 13.12, 0.37)	0.06
Th17	4	< 0.00001	Random	− 0.15 (− 0.57, 0.27)	0.48
Foxp3	2	0.09	Random	1.21 (− 0.58, 3.01)	0.19
Treg	3	< 0.00001	Random	4.73 (− 1.51, 10.97)	0.14
Renal sclerosis score	10	< 0.00001	Random	− 1.92 (− 2.66, − 1.18)	< 0.00001

### Assessment of ANA

Four experimental studies [10, 14, 18, 36] were recruited into this meta-analysis to detect the efficacy of MSC in lupus nephritis treatment. The results indicated that the MSC group obtained lower levels of ANA than the control group (OR = − 70.93, 95% CI − 104.55, − 37.32; *P* < 0.0001; Table 2).

### Assessment of Scr

The Scr levels were also assessed and eight studies [11–13, 17, 23, 24, 31, 37] were recruited. The results indicated that the difference in Scr levels between the MSC group and the control group was notable (OR = − 8.20, 95% CI − 12.71, − 3.69; *P* = 0.0004; Table 2), and the MSC group had lower levels of Scr.

### Assessment of BUN

Seven studies [11, 13, 16, 22–24, 37] were included to assess the effects of MSC on BUN. The results showed that the difference in BUN levels between the MSC group and the control group was notable (OR = − 14.57, 95% CI − 20.50, − 8.64; *P* < 0.00001; Table 2), and the MSC group had lower levels of BUN.

### Assessment of albumin

The albumin levels were also detected, and two studies [10, 23] were recruited. The results showed that the MSC group had higher levels of albumin, and the difference in albumin levels between the MSC group and the control group was notable (OR = 7.22, 95% CI 3.74, 10.69; *P* < 0.0001; Table 2).

### Assessment of proteinuria

**Fig. 4:**
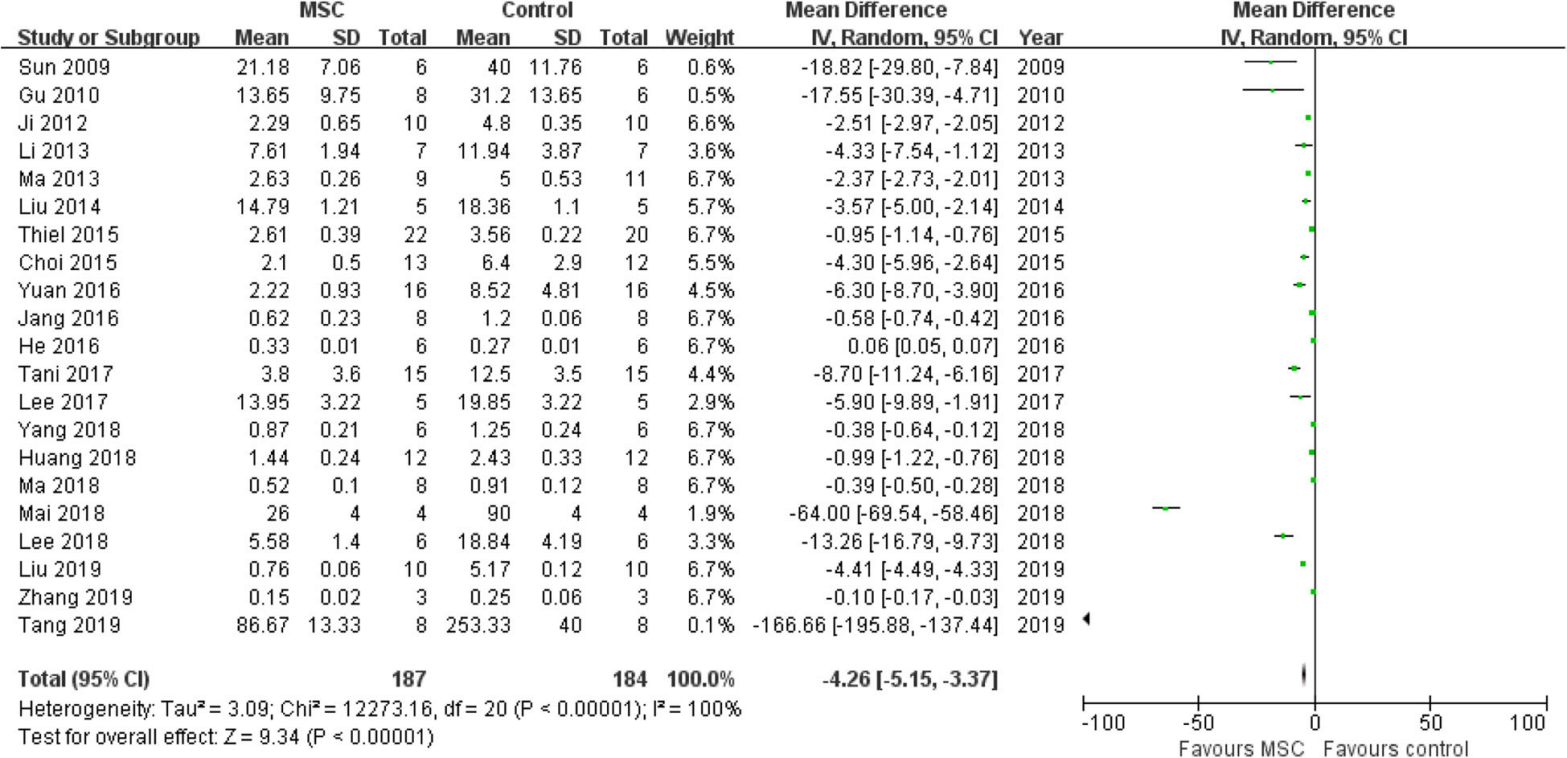
Assessment of proteinuria

Twenty-one studies [10, 11, 14–17, 20, 21, 23, 24, 27–35, 36, 37] were recruited into this meta-analysis for the assessment of MSC in reducing proteinuria. We found that the MSC group obtained a lower level of proteinuria when compared with the control group (OR = − 4.26, 95% CI − 5.15 to − 3.37; *P* < 0.00001; Table 2 and Fig. 4).

### Assessment of IgM

Three experimental studies [10, 19, 30] were identified in this meta-analysis to detect the effect of MSC on IgM in lupus nephritis treatment. The results showed that the difference in IgM levels between the MSC group and the control group was not notable (OR = − 4437.90, 95% CI − 12,581.07, 3705.28; *P* = 0.29; Table 2).

### Assessment of ILs

The levels of IL-2, IL-4, IL-6, IL-10, IL-12, and IL-17 were detected; four studies [12, 16, 20, 30] for IL-2, two studies [12, 20] for IL-4, seven studies [10, 12, 16, 20, 24, 36, 37] for IL-6, six studies [12, 16, 18, 20, 30, 36] for IL-10, three studies [12, 16, 20] for IL-12, and five studies [10, 20, 24, 25, 36] for IL-17 were included for the assessment of the effect of MSC treatment on ILs. Interestingly, the MSC treatment group had a lower level of IL-2, IL-12, and IL-17 when compared with the control group (IL-2: OR = − 50.86, 95% CI − 78.76, − 22.96; *P* = 0.0004; IL-12: OR = -328.24, 95% CI − 652.20, − 4.29; *P* = 0.05; IL-17: OR = − 36.40, 95% CI − 65.88, − 6.93; *P* = 0.02; Table 2). The levels of IL-4, IL-6, and IL-10 in the MSC group were lower than those in the control group, but the differences were not statistically significant (IL-4: OR = − 92.42, 95% CI − 332.33, 147.49; *P* = 0.45; IL-6: OR = -33.55, 95% CI − 83.31, 16.21; *P* = 0.19; IL-10: OR = − 29.67, 95% CI − 68.25, 8.91; *P* = 0.13; Table 2).

### Assessment of other cytokines

The levels of TGF-β, MCP-1, IFN-γ, TNF-α, Th1, Th17, Foxp3, and Tregs were detected; three studies [10, 18, 24] for TGF-β, two studies [11, 37] for MCP-1, four studies [12, 20, 24, 30] for IFN-γ, six studies [12, 16, 20, 24, 35, 37] for TNF-α, three studies [19, 23, 37] for Th1, four studies [19, 23, 26, 36] for Th17, two studies [10, 11] for Foxp3, and three studies [19, 23, 36] for Tregs were included for the assessment of the effect of MSC treatment on other cytokines. Interestingly, the MSC treatment group had a lower level of IFN-γ when compared with the control group (OR = − 240.24, 95% CI − 364.73, − 115.75; *P* = 0.0002; Table 2). The levels of TGF-β, MCP-1, TNF-α, Th1, and Th17 in the MSC group were lower than those in the control group, but the differences were not statistically significant (TGF-β: OR = − 0.09, 95% CI − 2.90, 2.72; *P* = 0.95; MCP-1: OR = − 5917.71, 95% CI − 17,303.66, 5468.23; *P* = 0.31; TNF-α: OR = − 74.71, 95% CI − 167.69, 18.28; *P* = 0.12; Th1: OR = − 6.37, 95% CI − 13.12, 0.37; *P* = 0.06; Th17: OR = -0.15, 95% CI − 0.57, 0.27; *P* = 0.48; Table 2). However, the levels of Foxp3 and Tregs in the MSC group were higher than those in the control group, but the differences were not statistically significant (Foxp3: OR = 1.21, 95% CI − 0.58, 3.01; *P* = 0.19; Treg: OR = 4.73, 95% CI − 1.51, 10.97; *P* = 0.14; Table 2).

### Assessment of renal sclerosis score

**Fig. 5:**
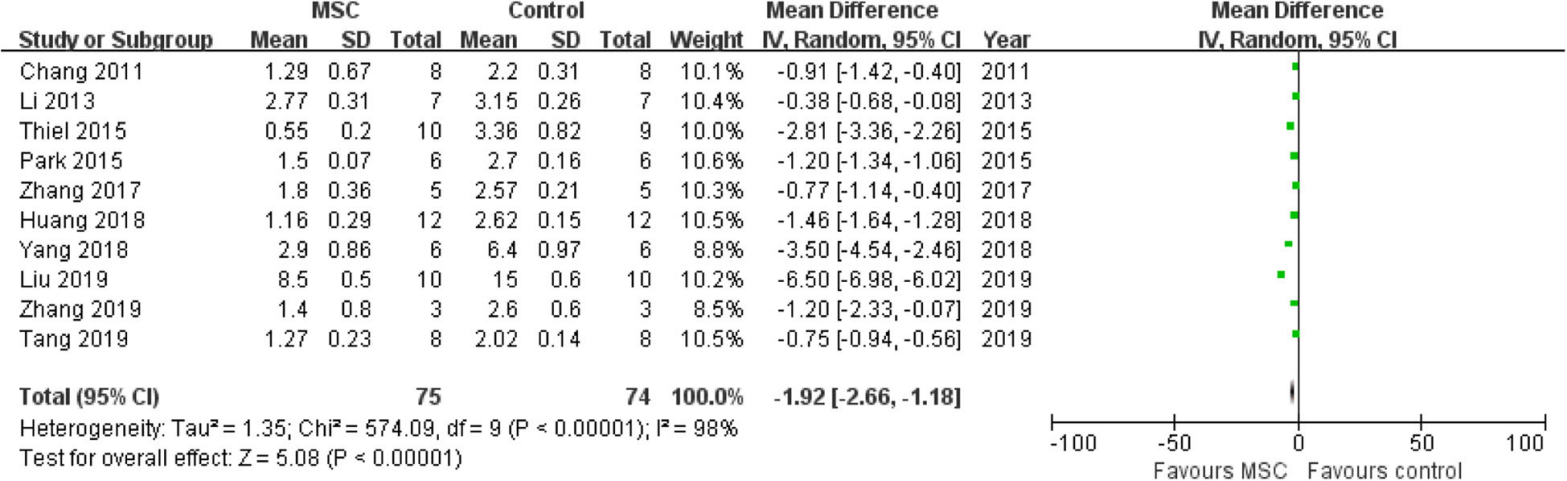
Assessment of renal sclerosis score

Ten studies [12, 16, 19, 26, 28, 32, 34, 35, 36, 37] were included for the assessment of the effect of MSC on renal sclerosis, and the renal sclerosis score was used. The results indicated that the MSC group had a lower renal sclerosis score when compared with the control group (OR = − 1.92, 95% CI − 2.66 to − 1.18; *P* < 0.00001; Table 2 and Fig. 5).

### Sensitivity analysis

We performed sensitivity analysis for the studies with a total number of test animals less than 16 and found that the MSC treatment group obtained a lower level of ds-DNA when compared with non-MSC treatment in lupus nephritis mice (OR = − 36.38, 95% CI − 52.46, − 20.30; *P* < 0.00001). When compared with the non-MSC treatment group, the MSC treatment group had a lower level of Scr (OR = − 5.97, 95% CI − 11.55, − 0.39; *P* = 0.04), BUN (OR = − 16.74, 95% CI − 23.77, − 9.70; *P* < 0.00001), and proteinuria (OR = − 3.46, 95% CI − 4.90, − 2.03; *P* < 0.00001) as well as a lower renal sclerosis score (OR = − 2.48, 95% CI − 3.98, − 0.97; *P* = 0.001).

### Publication bias

**Fig. 6:**
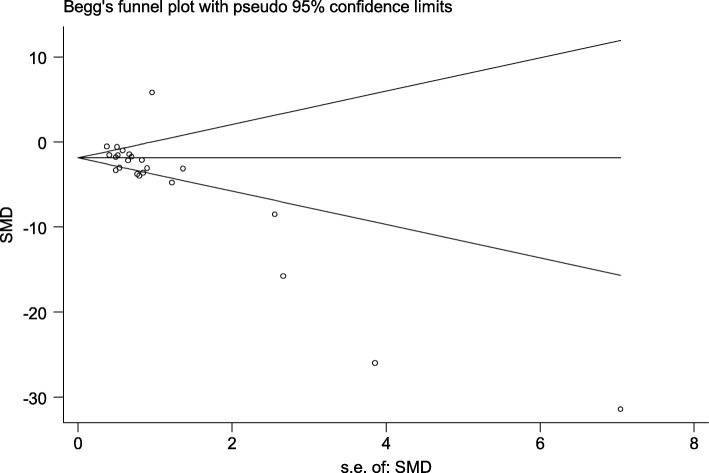
Publication bias

A funnel plot generated for the primary outcome using Egger’s test (*P* = 0.003) and Begg’s test (*P* = 0.002) suggested that there was publication bias (Fig. 6).

## Discussion

In this study, we included studies in mice, and the results might be more robust than those from clinical trials. In the past decades, glucocorticoids, cyclophosphamide, calcineurin inhibitors (cyclosporine A and tacrolimus), mycophenolate mofetil, rituximab, tripterygium wilfordii, etc., have been used in the treatment of lupus nephritis and were also mentioned in the KDIGO 2012 guidelines [38]. MSC has been reported to be a good agent for the treatment of some autoimmune diseases in the past decades [3, 39, 40].

MSC treatment resulted in lower levels of ds-DNA, ANA, Scr, BUN, proteinuria, and renal sclerosis score, and MSC treatment could get a higher level of albumin. These results indicated that MSC might be a good agent for the treatment of lupus nephritis in mice. To detect the potential, cytokines were also assessed, and we found that the MSC treatment group had lower levels of IL-2, IL-12, IL-17, and IFN-γ when compared with the control group. However, the difference was not notable for IL-4, IL-6, IL-10, TGF-β, MCP-1, TNF-α, Th1, Th17, Foxp3, and Tregs. The cytokines mentioned above might indicate that MSC treatment might play a protective role by regulating the signalling pathways of IL-2, IL-12, IL-17, and IFN-γ, but not IL-4, IL-6, IL-10, TGF-β, MCP-1, TNF-α, Th1,Th17, Foxp3, or Tregs. Lupus nephritis is a typical autoimmune disease characterised by the production of autoantibodies against nuclear antigens as well as renal involvement. Cytokines might take part in this process. The sample sizes for the meta-analyses were small, and more well-designed studies should be performed to confirm these findings.

In a previous study, there was only one meta-analysis assessing the efficacy of MSC in the therapy of kidney disease. Wang et al. [41] performed a meta-analysis including 21 studies to assess the efficacy of MSC treatment on renal failure and found that the elevated Scr level was reduced in the animal models with renal failure following MSC therapy. Furthermore, we also reviewed the systematic review and meta-analysis for assessing the efficacy of MSC treatment on autoimmune diseases. Liu et al. [42] included 48 studies to evaluate whether the MSC can improve the outcomes of rheumatoid arthritis, and the results indicated that MSC treatment consistently exhibited therapeutic benefits. Hynes et al. [43] performed a systematic review of 30 studies to investigate the evidence for the therapeutic efficacy of MSC treatment in arthritis and indicated that 19 demonstrated positive outcomes while 11 studies failed to demonstrate positive effects. There was no previous meta-analysis on the relationship between MSC treatment and lupus nephritis.

Quality assessment was performed in this meta-analysis. Most of the included studies were determined to have low or unclear risk of bias and were regarded as good quality. However, the publication bias test was performed and the result indicated publication bias. Most of the included studies lacked observer blinding, which will affect the robustness of the results. Furthermore, the sample size of most of the included studies was small (the total number of test animals was less than 16). Different types of MSC were included. These factors will affect the robustness of the results. More well-designed studies should be performed in the future. In this meta-analysis, we did not perform a meta-analysis for clinical trials because there might be less heterogenicity among studies with murine models of lupus kidney dysfunction for meta-analysis. The results in mice indicated that MSC treatment can have a good effect on lupus nephritis, and it indicated that more well-designed studies on MSC treatment for lupus nephritis in the clinic are needed in the future.

## Conclusions

In our meta-analysis, we found that MSC treatment resulted in lower levels of ds-DNA, ANA, Scr, BUN, proteinuria, and renal sclerosis score in lupus nephritis for mice, and MSC treatment could get a higher level of albumin. Our meta-analysis also indicated that the MSC treatment group also had lower levels of IL-2, IL-12, IL-17, and IFN-γ when compared with the control group in lupus nephritis mice. However, more studies are needed to confirm these associations in the future.

## Supplementary information

Additional file 1. The Preferred Reporting Items for systematic Reviews and Meta-Analyses (PRISMA) checklist.

## Data Availability

Not applicable.

## Abbreviations

CI: Confidence intervals
M-H: Mantel-Haenszel
MSC: Mesenchymal stem cells
SLE: Systemic lupus erythematosus
WMDs: Weighted mean differences
